# Extra-intestinal manifestations of Celiac disease in children: their prevalence and association with human leukocyte antigens and pathological and laboratory evaluations

**DOI:** 10.1186/s12887-022-03826-w

**Published:** 2023-01-04

**Authors:** Leila Salarian, Mohammad Khavaran, Seyed Mohsen Dehghani, Amirali Mashhadiagha, Seyed Ali Moosavi, Shayan Rezaeianzadeh

**Affiliations:** 1 Department of Pediatric Endocrinology, Shiraz University of Medical Sciences, Shiraz, Iran; 2 Shiraz Neonatal Research Center, Shiraz University of Medical Sciences, Shiraz, Iran; 3 Transplant Research Center, Shiraz University of Medical Sciences, Shiraz, Iran; 4 Student Research Committee, Shiraz University of Medical Sciences, Shiraz, Iran

**Keywords:** Celiac disease, Extra-intestinal manifestation, Human leukocyte antigen, Gluten-free diet

## Abstract

**Background:**

Celiac disease (CD) is an autoimmune disease caused by gluten intake. Traditionally CD was believed to be a disease of the gut, although a wide range of extra-intestinal manifestations (EIM) was recognized. The exact prevalence of EIM and the associated risk factors have not been well studied.

**Aim:**

We aimed to assess the prevalence of EIM in children with CD and their association with human leukocyte antigen (HLA) typing, and pathological and laboratory indices.

**Method:**

We conducted a cross-sectional study on children and adolescents with a definite diagnosis of CD. They were followed in the main Celiac Clinic of Southern Iran.

**Results:**

We included 204 children who were visited between 2012 and 2017. Nearly 85% of them were positive for HLA-DQ2 and 40.6% for HLA-DQ8. The most prevalent intestinal complaints reported were abdominal pain (42.6%) and chronic constipation (19.1%). Failure-to-thrive (32.7%), iron deficiency anemia (25%), short stature (20.5%), and eczema (18.6%) were the most common EIMs. However, failure-to-thrive and short stature were presented at significantly younger ages, whereas those patients with concomitant type 1 diabetes mellitus (DM) were significantly older. We also found significant relationships between autoimmune thyroid disease and HLA-DQ5, and the presence of headaches with HLA-DQ7. The prevalence of HLA types of DQ2, DQ8, DQ6, and DQ7 significantly varied among different Marsh groups. Patients who were positive for HLA-DQ8, were significantly older, taller, and weightier. No significant association was found between HLA types and any of the gastrointestinal symptoms, anti-tTG and compliance to gluten free diet. Moreover, there were no statistically significant differences detected between the presence of each individual EIM, the level of IgA anti-tTG, sex, and Marsh typing.

**Conclusion:**

This study highlights the presence of EIM in CD and their associated factors. We show the potential role of HLA typing in some EIMs, which may shed light for future studies.

## Introduction

Celiac disease (CD) is a complex autoimmune disease caused by gluten intake in genetically predisposed individuals. It results in high concentrations of celiac-specific autoantibodies and varying degrees of small intestine inflammation, as well as a wide spectrum of gastrointestinal- (GI), classical and extra-intestinal-, or non-classical symptoms [1].

Diarrhea, steatorrhea, abdominal pain, bloating, and weight loss are considered to be the most common complaints of patients with CD; these are due to maldigestion and malabsorption caused by intestinal villous atrophy. Constipation, frequent vomiting, or heartburn are less common and may lead to a misdiagnosis (e.g., a functional problem or irritable bowel syndrome) [2]. The manifestations of CD vary based on the population: Diarrhea, loss of appetite, abdominal distention (bloating), and failure-to-thrive are among the most common GI symptoms in children, including those younger than three years old [3], while adults and adolescents suffer more from diarrhea, bloating, constipation, abdominal pain, and weight loss [4].

Traditionally CD was believed to be a disease of the GI tract, although many extra-intestinal manifestations (EIM) symptoms were reported in the literature, and nowadays CD is considered to be a systemic disease. Growth retardation, short stature, delayed puberty, dental enamel hypoplasia, osteopenia/osteoporosis, iron-deficiency anemia refractory to oral iron supplementation, recurrent stomatitis, liver and biliary disease, dermatitis herpetiformis, arthralgia/arthritis are the most common EIM seen in the pediatric CD population [5, 6]. In addition, CD patients can exhibit a wide range of neurological symptoms such as headache, paresthesia, neuroinflammation, anxiety, and depression. Most of these symptoms can be reversed by a strict gluten-free diet (GFD), albeit fatigue and some neurological symptoms, as well as functional GI issues, may remain for a long time in a subset of CD patients [7]. Interestingly, the prevalence of CD EIM is similar in pediatric (60%) and adult (62%) CD, while the specific EIM and recovery rates vary. Short stature is the most prevalent EIM in children with CD, while iron deficiency anemia is the most common in adults. Fatigue and headaches are two of the more prevalent EIM in both children and adults. Compared to adults, children appear to improve at a much higher and faster rate [4, 7–13].

The human leukocyte antigen (HLA) plays a role in CD pathogenesis. HLA-DQ2 and -DQ8 haplotypes have been suggested as the main genetic risk factors since the development of CD is very rare without one of them. Approximately 40% of individuals carry one or both of these HLAs, but only a fraction of them will go on to develop CD. Moreover, negative associations between HLA DQ5 and DQ6 and CD were reported in a previous study, while in another investigation, HLA DQ7 found to be an additive or independent CD risk haplotype further than DQ2 and DQ8. HLA risk has been associated to various CD-related features, such as age at onset, clinical outcomes, antibody levels, and grade of histological lesion [14–18].

Although several EIMs in CD are well recognized, their true prevalence is not known, nor whether the existence of such symptoms influences the severity of histological damage or other CD characteristics at the time of diagnosis. The vast majority of CD patients are still either undiagnosed or have a long delay before diagnosis [6]. Better knowledge of EIM will improve diagnostic efficacy and avoid unnecessary hardship for patients, as well as potentially severe complications associated with undiagnosed/untreated CD. We aimed to study the prevalence of EIM and their association with HLA typing, and pathological and laboratory indices in children with CD.

## Method

### Design and population

A cross-sectional study was conducted on children and adolescents with a definite diagnosis of CD who were being followed in the Imam Reza Clinic, which is affiliated to Shiraz University of Medical Sciences. CD was diagnosed through clinical manifestations and confirmed with serological and histopathological findings. A titer of 10 IU/mL or higher for anti-tissue transglutaminase IgA (anti-tTG) and Marsh type 2 or more severe types in the histological evaluation was considered confirmatory for CD.

### Data collection

Demographic data (age, sex), anthropometric measures (height, weight at time of diagnosis), as well presenting clinical manifestations (diarrhea, constipation, abdominal distention, bloating), levels of IgA anti-tTG, histopathological findings based on Marsh classification, and HLA types (DQ2, DQ8, DQ5, DQ6, and DQ7) were collected from patients’ medical records retrospectively. HLA genotyping tests were performed by either polymerase chain reaction (PCR/RFLP) method or flowcytometry on the blood samples. Due to the limited availability of HLA typing tests, it is not mandatory for patients in the Celiac clinic, indeed this data was collected only for some patients.

Endocrinological (diabetes mellitus, autoimmune thyroid disease, short stature, and delayed puberty), dermatological (dermatitis herpetiformis, acquired ichthyosis, cutaneous vasculitis, eczema, and postural dermatitis), and neuro-psychological manifestations (hypotonia, developmental delay, learning disabilities, headache, peripheral neuropathy, ataxia, dysthymia, epilepsy, anxiety, and depression), as well as dental enamel defects, metabolic bone diseases, arthritis, liver disease, and iron deficiency were considered to be the most important EIM. These clinical signs and symptoms were checked by a physician in the celiac clinic routinely and were also gathered from the medical records at the time of study.

Short stature and failure-to-thrive are defined as a height and weight below the 3^rd^ percentile of the (national) growth curve at the time of diagnosis. Iron deficiency anemia is considered if hemoglobin and ferritin are lower than normal ranges for age and sex. Any increase in hepatic transaminases was labelled as a hepatic involvement. Metabolic bone diseases were considered based on positive densitometric findings of the spine and femoral head. Anxiety and depressive disorders were diagnosed via the Diagnostic and Statistical Manual of Mental Disorders’ criteria (DSM-5).

Patients were also divided into three groups based on gluten-free diet compliance: good (normal antibody titer), fair (antibody titer up to three times normal), and poor (antibody titer more than three times normal).

### Statistical analysis

The data were analyzed using Statistical Package for Social Sciences (SPSS) version 16.0 (IBM, Armonk, NY, USA). Descriptive analysis was used for demographic variables; frequencies and percentages were estimated for categorical variables. Independent samples were recruited to use in paired T-tests and Chi-square tests to analyze the relationship between EIM, respectively, with age and antibody titer, and Marsh type with HLA, gender, and dietary compliance.. The Mann–Whitney test was used to analyze data on bone metabolic diseases. Fisher’s Exact test was used to investigate the relationship of HLA types with sex, compliance, and clinical symptoms. Independent samples t-test was utilized in order to analyze the relationship of age and HLA types. Mann–Whitney U test was utilized to analyze the relation between HLA types and TTG Titer, Weight, and height. A two-tailed test (*p*-value < 0.05) was considered statistically significant.

## Results

A total of 204 eligible children who were visited at CD clinic from April 2012 to April 2017 were studied. Participants were between 1–18 years old, with a mean of 8.11 ± 3.41 years; 127 (62.2%) cases were female; 16 (7.8%) reported a positive family history for CD. There were also 38 (18.6%) cases of DM, 10 (4.9%) cases of autoimmune thyroid disease, and 1 case of autoimmune hepatitis among the studied population.

The most common GI symptoms were abdominal pain 87 (42.6%), chronic constipation 39 (19.1%), bloating 32 (15.7%), and chronic diarrhea 34 (16.7%). Failure-to-thrive was seen in 52 (32.7%) cases and 32 (20.5%) had short stature. There were no cases of diagnosed delayed puberty in this group. The available laboratory results revealed 27 (25%) cases of iron deficiency anemia (2%) cases diagnosed with metabolic bone disease; and 4 (2%) cases with raised hepatic transaminase level. There were no cases of arthritis or defects in dental enamel.

Dermatological examinations revealed 38 (18.6%) cases of eczema and 23 (11.3%) cases of hair loss. There were no cases of vitiligo, herpetiform dermatitis, acquired ichthyosis, cutaneous vasculitis, or pustular dermatitis in our population.

There was only one (0.5%) case of epilepsy, while 7 (3.4%) cases reported headache among neurological manifestations; 12 (5.9%) cases fulfilled anxiety disorder criteria; and one (0.5%) was diagnosed with depression. Our results for GI and EIM in CD in our study population are summarized in Table 1.

**Table 1:** Gastrointestinal and extraintestinal manifestations of celiac disease, pathological findings, and human leukocyte antigen (HLA) classifications in our pediatric population

	Frequency (percentage)
Gastrointestinal manifestations	
Abdominal pain	87 (42.6%)
Chronic constipation	39 (19.1%)
Bloating	32 (15.7%)
Chronic diarrhea	34 (16.7%)
Extra intestinal manifestations	
Developmental	
Failure to thrive	52 (32.7%)
Short stature	32 (20.5%)
Delayed puberty	0 (0%)
Hematologic	
Iron deficiency anemia	27 (25%)
Hepatological	
Elevated transaminases	4 (2%)
Dermatological	
Eczema	38 (18.6%)
Alopecia	23 (11.3%)
Herpetiform dermatitis	0 (0%)
Others	0 (0%)
Neuropsychiatric	
Seizure	1 (0.5%)
Headache	7 (3.4%)
Anxiety	12 (5.9%)
Depression	1 (0.5%)
Others	0 (0%)
Miscellaneous	
Dental enamel defects	0 (0%)
Arthritis	0 (0%)
Marsh Classification	
1	2 (1.3%)
2b	6 (3.8%)
3a	51 (31.9%)
3b	59 (36.9%)
3c	42 (26.3%)
HLA typing	
DQ2	60 (85.7%)
DQ8	28 (40.6%)
DQ6	6 (9%)
DQ5	18 (26.9%)
DQ7	9 (13.4%)
Compliance to gluten-free diet	
Good	77 (37.3%)
Fair	58 (28.4%)
Poor	369 (33.8%)

The range of IgA anti-tTG was between 0.9–1403, with a mean of 214.7 ± 229.04 IU/ml. Based on levels of IgA anti-tTG at their last visit, patients were classified into three classes to indicate compliance with a GFD: 77 (37.7%) were in the compliant group, 58 (28.4%) in the fair group, and 69 (33.8%) in the poor group.

Marsh classification of duodenal biopsies revealed type 3B (36.9%) as the most common, followed by 3A (31.9%). We performed HLA typing for 70 cases and detected HLA-DQ2 in 60 of them; this was the most prevalent HLA type. Marsh classifications and frequencies for HLA types are given in Table 1.

The T-test revealed a statistically significant relation between age and failure-to-thrive (*p*-value < 0.001), as well as short stature (*p*-value < 0.001). Both presented at significantly younger ages. Those with concomitant DM were statistically significant older (*p*-value = 0.016). The results are shown in Table 2.

**Table 2:** Association between age and some of extra-intestinal manifestations of Celiac disease

Clinical manifestation		Age (year, mean (SD))	*P*-value
Failure to thrive	Presence	6.78 (3.25)	< 0.001
	Absence	8.94 (3.11)	
Short stature	Presence	6.25 (2.40)	< 0.001
	Absence	8.75(3.45)	
Diabetes mellitus	Presence	9.31 (3.73)	0.016
	Absence	7.83 (3.29)	
Autoimmune thyroid diseases	Presence	8.80 (3.79)	0.56
	Absence	8.07 (3.40)	

In both males and females, there were significantly more HLA-DQ7 negative individuals than HLA-DQ7 positive and HLA-DQ7 is more frequently associated with female gender (*p*-value = 0.021). The prevalence of HLA types significantly varied among different Marsh groups except for HLA-DQ5. HLA types of DQ2, DQ8, and DQ7 are associated with histological lesions belonging to Marsh 3a class, while DQ6 is more frequently detected in Marsh 3c class. The *p*-values for DQ2, DQ8, DQ6, and DQ7, were 0.008, 0.032, 0.027, and 0.003 respectively. Patients who were positive for HLA-DQ8, were significantly older than HLA-DQ8 negative individuals (*p*-value = 0.016). There was also a statistically significant relation between HLA-DQ8 and both weight and height (*p*-values = 0.005, and 0.038 respectively). No significant association was found between HLA types and any of the gastrointestinal symptoms (including chronic diarrhea, chronic constipation, abdominal pain, abdominal distension and bloating), anti-tTG and compliance to gluten free diet. Moreover, there is a significant relationship between autoimmune thyroid disease and HLA-DQ5, and the presence of headache with HLA-DQ7 (*p*-value = 0.011). There were no statistically significant differences between the presence of each examined EIM of CD, level of IgA anti-tTG, sex, or Marsh typing (*p*-value > 0.05). Our findings are presented in the Table 3.

**Table 3:** The association between age, sex, Marsh classifications, weight and height, IgA anti-tTG and some of extra-intestinal manifestations and HLA types of children with Celiac disease

	**DQ2**			*P -value*	**DQ8**		*P -value*	**DQ6**		*P -value*	**DQ5**		*P -value*	**DQ7**		*P -value*
	**Pos** N (%) or Mean (SD)		**Neg** N (%) or Mean (SD)		**Pos** N (%) or Mean (SD)	**Neg** N (%) or Mean (SD)		**Pos** N (%) or Mean (SD)	**Neg** N (%) or Mean (SD)		**Pos** N (%) or Mean (SD)	**Neg** N (%) or Mean (SD)		**Pos** N (%) or Mean (SD)	**Neg** N (%) or Mean (SD)	
**Sex**				1.000			0.433			0.664			0.464			**0.021**
Male	22 (31.4)		4 (5.7)		9 (13.0)	17 (24.6)		3 (4.5)	22 (32.8)		8 (11.9)	17 (25.4)		0 (0.0)	25 (37.3)	
Female	38 (54.3)		6 (8.6)		19 (27.5)	24 (34.8)		3 (4.5)	39 (58.2)		10 (14.9)	32 (47.8)		9 (13.4)	33 (49.3)	
**Marsh Class**				**0.008**			**0.032**			**0.027**			0.702			**0.003**
2b	1 (1.6)		1 (1.6)		2 (3.2)	0 (0.0)		0 (0.0)	2 (3.2)		1 (1.6)	1 (1.6)		1 (1.6)	1 (1.6)	
3a	23 (35.9)		2 (3.1)		10 (15.9)	14 (22.2)		1 (1.6)	23 (37.1)		8 (12.9)	16 (25.8)		4 (6.5)	20 (32.3)	
3b	21 (32.8)		3 (4.7)		9 (14.3)	15 (23.8)		1 (1.6)	22 (35.5)		6 (9.7)	17 (27.4)		2 (3.2)	21 (33.9)	
3c	9 (14.1)		2 (3.1)		1 (1.6)	10 (15.9)		4 (6.5)	7 (11.3)		2 (3.2)	9 (14.5)		0 (0.0)	11 (17.7)	
**IgA Anti-tTG**^a^	250.13 (232.34)		317.49 (310.34)	0.681	293.18 (261.10)	243.44 (234.71)	0.588	205.62 (168.16)	268.30 (253.75)	0.747	258.61 (258.89)	263.92 (244.64)	0.754	185.99 (163.75)	273.34 (255.55)	0.423
**Age**	8.03 (3.05)		10.10 (4.07)	0.063	9.50 (3.40)	7.59 (2.97)	**0.016**	9.33 (2.07)	8.31 (3.36)	0.469	8.33 (3.83)	8.43 (3.08)	0.917	8.11 (2.80)	8.45 (3.36)	0.776
**Weight**^a^	26.60 (17.79)		33.80 (19.99)	0.100	35.97 (25.97)	22.45 (7.13)	**0.005**	29.50 (9.59)	27.56 (19.14)	.196	25.64 (15.54)	28.55 (19.51)	0.519	25.62 (6.93)	28.08 (19.67)	0.798
**Height**^a^	122.45 (21.59)		132.40 (28.32)	0.098	129.60 (28.46)	120.38 (17.94)	**0.038**	130.17 (19.93)	123.18 (23.39)	0.383	122.22 (23.83)	124.44 (22.97)	0.692	125.67 (14.52)	123.53 (24.22)	0.827
**Extra-intestinal manifestations**																
**Headache**				0.681			0.223			0.752			0.541			**0.011**
Presence	1 (1.4)	0 (0.0)			1 (1.4)	0 (0.0)		0 (0.0)	1 (1.5)		0 (0.0)	1 (1.5)		1 (1.5)	0 (0.0)	
Absence	59 (84.3)	10 (14.3)			27 (39.1)	41 (59.4)		6 (9.0)	60 (89.5)		18 (26.9)	48 (71.6)		8 (11.9)	58 (86.6)	
**Autoimmune thyroid disease**				0.528			0.513			0.246			**0.025**			0.417
Presence	3 (4.3)		1 (1.4)		1 (1.5)	3 (4.3)		1 (1.5)	3 (4.5)		3 (4.5)	1 (1.5)		0 (0.0)	4 (6.0)	
Absence	56 (81.4)		9 (12.9)		27 (39.1)	38 (55.1)		5 (7.5)	58 (86.5)		15 (22.4)	48 (71.6)		9 (13.4)	54 (80.6)	

^a^Means and Standard Deviations were mentioned for easier interpretation of results even though Mann–Whitney U test was used instead of independent samples t-test due to non-normal data

## Discussion

In our study, we found abdominal pain and chronic constipation were the most common GI symptoms, while failure-to-thrive, iron deficiency anemia, and short stature were the most common non-GI symptoms. These manifestations are consistent with other studies [19–21]. This is the first study in which a statistically significant relationship between age and failure-to-thrive, as well as age and short stature; both appeared at significantly younger ages. Furthermore, those with concomitant diabetes were significantly older. These findings are important because timely prevention and treatment can be beneficial to patients’ well-being and development, and it is preferable to adopt treatment, in the form of a strict gluten-free diet, before puberty. It is recommended that children diagnosed with CD be carefully evaluated for both failure-to-thrive and short stature from an early age and that their condition is monitored regularly for necessary interventions.

In our study, 85.7% of patients were positive for HLA-DQ2 and 40.6% for HLA-DQ8. This result is consistent with a previous study on an Iranian population, which concluded that HLA-DQ2 and -DQ8 were carried by 80% and 49% of CD patients, respectively [22]. The high frequency of DQ8 has also been reported in American Indians (25.3%), South Americans (28.3%), and Middle Easterns (22%) [23]. However, results from European studies showed that approximately 86–93% of CD patients carried DQ2 variants, while a minority of them also carried DQ8 [16, 24, 25]. In another European multicenter study, it was observed that DQ2 was present in approximately 86–93% of CD patients, while around 3–8% of the study’s patients had DQ8 without DQ2 [26]. In our study we show that the frequency of DQ8 in our population is higher than that reported by European studies, but it is close to the frequencies found in South America and the Middle East [23]. These results suggest that the higher prevalence of the HLA-DQ8 type in Iranian CD patients is similar to other non-European patient groups, and this inconsistency should be a focus of future studies.

In terms of CD’s association with other autoimmune diseases, we found no cases of vitiligo and alopecia among our 204 patients, although 18.6% of them had diabetes, and 4.9% had autoimmune thyroiditis. The association between type 1 diabetes and CD comes from the autoimmune nature of these diseases. Hummel et al. reported a 3.5% prevalence of CD among diabetic children [27]. Similarly, the association between CD and autoimmune thyroid disease has been demonstrated in previous studies in pediatric populations and is three times higher than in the general population [28]. Another study conducted on children with CD showed a prevalence of autoimmune thyroid disease of 10.5%, which is higher than our finding [29]. Based on previous studies, 2–7% of patients with autoimmune thyroiditis will eventually go on to develop CD [28–32].

Headaches are the most common neurological symptom detected in children with CD, although the exact mechanism by which CD causes headaches is unknown. However, it is thought to be secondary to vitamin- and macro-element deficiencies and low serotonin levels, all due to CD-related malabsorption [5]. There is little evidence for the relation between HLA-DQ7 and headache, or HLA-DQ5 and autoimmune thyroid disease in the literature; however previous studies on HLA and CD manifestations have found that genetics play an important role [33].

Our results showed that individuals with HLA-DQ8 were significantly older at the time of diagnosis than those who did not carry HLA-DQ8. There was also a statistically significant relation between HLA-DQ8 and both weight and height in the studied population which is because of two-year age difference of these group (reflecting age difference). This may concordant with a previous study in which has been linked to as age at onset of CD and HLA typing [14], however, in a recent study failed to link a relation between age of onset, sex, Marsh class and HLA types, while hypertransaminasemia and levels of anti-tTG were significantly affected by HLA typing [34]. There was not any statistically significant association between HLAs and anti-tTG titer in our population.

There was not any significant association between HLA types and GI symptoms including chronic diarrhea, chronic constipation, abdominal pain, abdominal distension and bloating in our study. These results are consistent with a review on the effects of HLA in CD and disease manifestations. The study revealed that studies in siblings largely reject the influence of HLA status on clinical manifestations, and instead point to environmental or non-HLA genetic factors as determining possible underlying clinical differences [14]. In addition, a recent study of 66 CD-positive sibling pairs found low clinical concordance between relatives in malabsorption or anemia, GI symptoms, EIM, and asymptomatic CD. Since HLA was concordant in 70% of the sibling pairs and clinical discordance was seen in 74% of the pairs, a higher proportion of similar genetic factors was seen in the pairs with discordant clinical presentation [15]. However, Martinez-Ojinaga et al. found that HLA-DQ genotypic frequencies differ between CD patients depending on their family history of CD. Notably, patients without first-degree relatives with CD seem to have a more classic clinical presentation and more severe histological damage when carrying HLA-DQ2.5 [33]. A review of the effects of HLA in CD and disease manifestations revealed that studies in siblings largely reject the influence of HLA status on clinical manifestations and instead point to environmental or non-HLA genetic factors as possible underlying clinical differences [14]. However, more research is needed to determine the exact correlation between different HLA types in this population and their relation to the frequency and severity of symptoms.

In terms of Marsh classification, nearly 98% of patients in our current study had Marsh class 2/3. Our findings are consistent with a review of data by the European Society of Pediatric Gastroenterology, Hepatology, and Nutrition Guidelines for Diagnosing CD 2020, which included 555 asymptomatic children with IgA anti-tTG titers 10 × upper limit of normal and who had diagnostic small bowel biopsies, with 94.2% having Marsh class 2 or 3 duodenal lesions [35]. In our study, most of studied HLA types including DQ2, DQ6, DQ8 are significantly associated with the histopathological classification of CD at the time of diagnosis which is concordant with previously mentioned review and inconsistent with a recent study [33, 34]. However, we found no significant difference between the presence of EIM in CD and compliance with a GFD, sex, Marsh class, or levels of IgA anti-tTG. Our result is consistent with a study of 71 children with CD, which concluded that, apart from chronic fatigue in patients on a partial diet, they did not differ significantly in the frequency of symptoms compared to the group who had high compliance with a GFD [36]. Although some authors report GI symptoms, fatigue, headache, and sleep disorders in patients with CD who are not on a strict diet, the majority of these patients had no symptoms [37–41].

Every retrospective design faces some inherent limitations base on its nature. Some mild symptoms, like early stages of tooth enamel defects did not get much attention from the patients and were also missed by their parents and physicians. Some of the EIM in CD, like depression, are subjective and difficult to assess in a pediatric population. Some other symptoms were presented as the disease progressed, especially in those with an untreated condition or poor compliance with a GFD. There is also a selection bias since we surveyed those who attended regular follow-ups in our CD clinic.

## Conclusion

This study highlights the presence of EIM in CD and their associated factors. We show the potential role of HLA typing in some EIMs, and also its relation to histopathological lesions and age of onset for CD with HLA DQ8. These may shed light for future studies and better understanding EIM and role of HLAs in physiopathology, and individualized treatment potentials of CD.

## Data Availability

The datasets analyzed during this study are not publicly available due to privacy and ethical restrictions but are available from the corresponding author on reasonable request.

## Abbreviations

anti-tTG: Anti-tissue transglutaminase IgA
CD: Celiac disease
DM: Diabetes mellitus
EIM: Extra-intestinal manifestations
GI: Gastrointestinal
HLA: Human leukocyte antigen
GFD: Gluten-free diet
